# Perimeter Control Method of Road Traffic Regions Based on MFD-DDPG

**DOI:** 10.3390/s23187975

**Published:** 2023-09-19

**Authors:** Guorong Zheng, Yuke Liu, Yazhou Fu, Yingjie Zhao, Zundong Zhang

**Affiliations:** Beijing Key Lab of Urban Intelligent Traffic Control Technology, North China University of Technology, Beijing 100144, China; zhengguorong@ncut.edu.cn (G.Z.); 1113503565@mail.ncut.edu.cn (Y.F.); zhaoyingjie@mail.ncut.edu.cn (Y.Z.); zdzhang@ncut.edu.cn (Z.Z.)

**Keywords:** perimeter control, macroscopic fundamental diagram, deep reinforcement learning

## Abstract

As urban areas continue to expand, traffic congestion has emerged as a significant challenge impacting urban governance and economic development. Frequent regional traffic congestion has become a primary factor hindering urban economic growth and social activities, necessitating improved regional traffic management. Addressing regional traffic optimization and control methods based on the characteristics of regional congestion has become a crucial and complex issue in the field of traffic management and control research. This paper focuses on the macroscopic fundamental diagram (MFD) and aims to tackle the control problem without relying on traffic determination information. To address this, we introduce the Q-learning (QL) algorithm in reinforcement learning and the Deep Deterministic Policy Gradient (DDPG) algorithm in deep reinforcement learning. Subsequently, we propose the MFD-QL perimeter control model and the MFD-DDPG perimeter control model. We conduct numerical analysis and simulation experiments to verify the effectiveness of the MFD-QL and MFD-DDPG algorithms. The experimental results show that the algorithms converge rapidly to a stable state and achieve superior control effects in optimizing regional perimeter control.

## 1. Introduction

Traffic problems have always been troubling in terms of urban governance and affect the economic development of cities. The continuous development of traffic information detection technology has improved the accuracy and timeliness of collected traffic data, making it increasingly clear to describe traffic congestion phenomena. For example, according to the “2021 Traffic Analysis Report on Major Chinese Cities”, compared to 2020, 60% of China’s 50 major cities have seen an increase in peak travel delays [1].

For a long time, reducing the occurrence and alleviating the impact of traffic congestion have been topics of concern for researchers in the transportation field. Therefore, in many research fields related to traffic congestion, research progress has been made in the analysis, modeling, prediction, and control of traffic congestion phenomena, such as traffic flow theory, traffic planning, traffic control, and intelligent transportation systems. Prior studies have proposed various methods to address the perimeter control problem based on the Macro Fundamental Diagram (MFD) [2]. Among these methods, model predictive control (MPC) [3] has shown promise and is widely used. However, the success of forecasting methods heavily relies on accurate forecasting models. Although network MFD estimation has been extensively studied [4], the scarcity of empirically observed MFDs in the literature highlights the practical challenges in estimating such MFDs. MPC, as a rolling-level control scheme, may not generalize well to real-world scenarios due to its sensitivity to level parameters and modeling uncertainties [5]. Non-MPC methods for perimeter control have also been proposed and proven effective. These include proportional integral-based control [6], adaptive control [7], and linear quadratic regulators [8]. However, all of these methods are model-based (i.e., assuming prior knowledge of the traffic dynamics of the entire region) or require information about the network’s MFD, making the models susceptible to potential errors between the predictive model and the actual environment dynamics.

However, the overall traffic control still relies mainly on traditional modes at different levels of control. As the transportation system is a complex system, it is difficult to achieve the overall traffic optimization effect of the entire transportation system by only pursuing the maximum traffic benefits of a single or multiple intersections with control objectives. Therefore, it is necessary to consider higher-level traffic area control, such as perimeter control, to obtain optimal traffic control effects for the entire system.

## 2. Model Construction

The MFD-QL model for perimeter control is constructed by combining the Q-learning algorithm [9] in reinforcement learning. It incorporates traffic information from the macroscopic fundamental diagram (MFD) into the perimeter control model, allowing for traffic optimization through adjustments to the perimeter control. Similarly, the MFD-DDPG model for border control is constructed by combining the DDPG algorithm [10] in reinforcement learning. It also incorporates traffic information from the MFD in the border control model. The MFD-DDPG border control model mitigates the impact of the information explosion in the traffic environment obtained from the DDPG algorithm, thereby achieving traffic optimization goals.

### 2.1. MFD-QL Perimeter Control Model

The MFD-QL model is a perimeter control model that incorporates feedback design for the overall traffic area. It utilizes the basic traffic flow information obtained from the traffic environment and captures real-time changes in the MFD within the traffic area. The MFD serves as a valuable tool for representing the traffic area information and assessing the traffic status, providing crucial information for further research and traffic feedback control. Table 1 presents the basic traffic information available for a traffic area.

By extracting the traffic elements from the traffic environment, we can obtain the traffic status of the traffic area. For a specific traffic area i, it is associated with an internal link collection $S_{i}$ and the lengths $l_{k}$ of each link k. By summing up the lengths of all links within the traffic area, we can obtain the total length $L_{i}$, as shown in Equation (1):

(1) $$L_{i}=\sum_{k\in U_{i}}l_{k}$$

Similarly, when considering the traffic volume $n_{k}(t)$ on link k, it is necessary to calculate the weighted traffic flow of the entire transportation area, as shown in Formula (2):

(2) $$Q_{i}(t)=\frac{\sum_{k\in U_{i}}l_{k}n_{k}(t)}{L_{i}}=\frac{\sum_{k\in U_{i}}l_{k}n_{k}(t)}{\sum_{k\in U_{i}}l_{k}}$$

The traffic density of the entire traffic area can be calculated using Formula (3):

(3) $$D_{i}(t)=\frac{\sum_{k\in U_{i}}n_{k}(t)}{L_{i}}=\frac{\sum_{k\in U_{i}}n_{k}(t)}{\sum_{k\in U_{i}}l_{k}}$$

The weighted average traffic speed of the traffic area can be obtained using Formula (4):

(4) $$V_{i}(t)=\frac{dQ_{i}(t)}{dD_{i}(t)}=\frac{\sum_{k\in U_{i}}l_{k}n_{k}(t)\sum_{k\in U_{i}}l_{k}}{\sum_{k\in U_{i}}l_{k}\sum_{k\in U_{i}}n_{k}(t)}=\frac{\sum_{k\in U_{i}}l_{k}n_{k}(t)}{\sum_{k\in U_{i}}n_{k}(t)}$$

Thus, the overall traffic information of the traffic area can be obtained, and the MFD of the traffic area can be further obtained in the traffic environment, thereby obtaining the traffic status of the traffic area.

Because the transportation system is a system that dynamically changes over time, continuous control is also required for the traffic control strategies within it to achieve significant traffic benefits.

**Figure 1:**
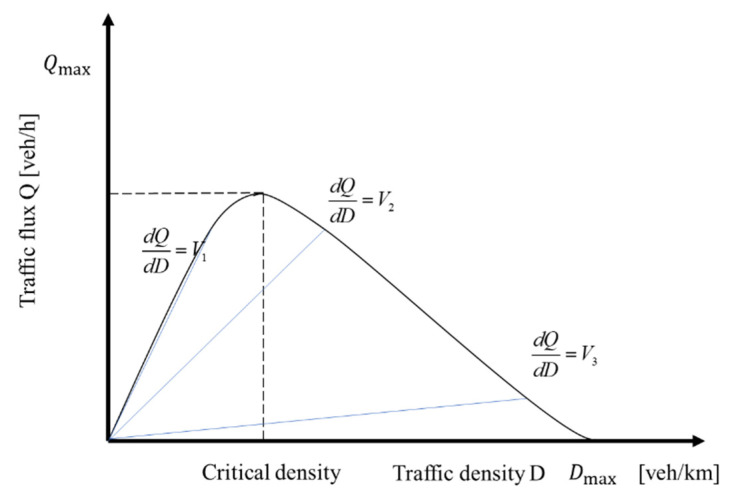
Traffic income division of MFD.

According to Figure 1, it can be concluded that there is a high traffic income interval in the traffic status of the transportation area, and using this as the critical value, the overall traffic status can be divided into two parts: the traffic unsaturated state and the traffic saturated state.

Using traffic speed as a physical quantity to characterize the traffic benefits of a transportation area, the traffic benefits of the area can be obtained based on the interval of the average speed of the traffic flow within the area, as shown in Formula (5):

(5) $$H_{i}(t)=\left\{\begin{array}{c} h_{i1},V_{i}(t)\in \left[V_{cri},V_{f}\right]\\ h_{i2},V_{i}(t)\in \left[V_{cri},V_{0}\right] \end{array}\right.$$

The value function of the Q-learning algorithm in the MFD-QL model incorporates the MFD parameters obtained from the traffic environment and modifies the traffic reward based on the traffic status of the traffic area. The modified value function can be represented as Formula (6):

(6) $$R_{i}(t)=\left\{\begin{array}{c} r_{i1},h_{i}\in h_{i1}(t)\\ r_{i2},h_{i}\in h_{i2}(t) \end{array}\right.$$

**Figure 2:**
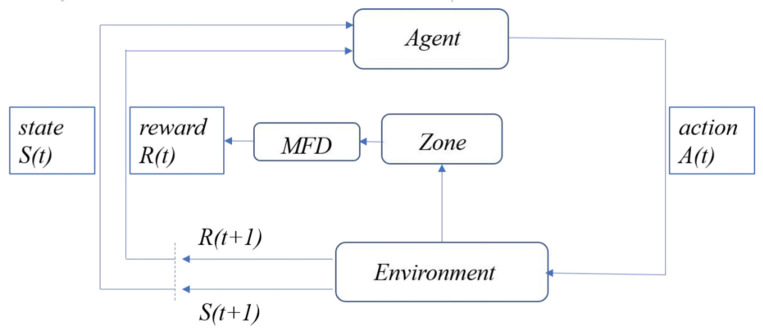
MFD-QL control framework diagram.

The control framework diagram of MFD-QL algorithm is shown in Figure 2.

The algorithm flow of MFD-QL is shown in Algorithm 1.
**Algorithm 1:** MFD-QL algorithm**Input:** Learning rate α, discount factor γ, number of iterations E, iteration step size T, *Inititalize* Q(s,a) is any value**for** e=l,…,E do  Initialization status s  **for** step=0 to T **do**    Select action a in state ε-greedy based on strategy s    Execute action a to obtain the next state s_t+1_    Calculate reward value r through MFD theory and environmental feedback    Update Q: Q(s,a)←Q(s,a)+a[r+ymaxaQ(s_t+1_,a_t+1_)-Q(s,a)]    s←s_t+1_
  **end**
**end**

Firstly, the traffic simulation environment is initialized and the set traffic environment parameters are imported, all agents are initialized, and the initial state of the agents is obtained from the traffic environment. The learning process of whether all agents are learning the MFD-QL algorithm at this time is begun. If the agent does not need to learn, it will cross the agent. If the agent needs to learn, it will use a greedy strategy ε to randomly select actions in the action space. The probability of selecting actions with the maximum Q value is 1−ε. After all agents have selected and executed actions, all agents obtain a new state, and only those who have learned receive rewards. The Q value and Q table of the intelligent agent are updated, and the learning process is complete. Then, the next learning can begin.

### 2.2. MFD-DDPG Perimeter Control Model

The MFD-DDPG perimeter control model is an extension of the MFD-QL model that addresses the limitations of discrete strategies in reinforcement learning for traffic signal control. It introduces a continuous control scheme to enable the fine control of the perimeter controller by choosing flexible perimeter control values.

In the MFD-DDPG algorithm, the agent interacts with the environment to gather experiences, and a distributed architecture is used for efficient data generation. The learning algorithm contains a large number of data simulation generators and a single centralized learner. Each generator has its own environment and assigns different values for the exploration strategy, which are stored in a fixed-range replay buffer according to the order in which the replay buffer is updated when it is saturated with values, which ensures that the source of experience is the most recent learning exploration strategy. The centralized learner draws experience samples from the shared replay buffer for updating the neural network of the intelligentsia in the network.

The MFD-DDPG algorithm flowchart is shown in Algorithm 2.
**Algorithm 2:** MFD-DDPG algorithm**Input:** Number of iterations E, iteration step size T, experience playback Set D, Sample size m, discount factor γ, Inititalize {Current network parameters θ^Q^ and θ^Q’^} Inititalize {Target network parameters θ^u^ and θ^u’^} Inititalize {Clear Experience Playback Collection D}**for** e=l, …, E do  Obtain the initial state s_t_ and random noise sequence N for action selection  **for** t=l, …, T **do**    Based on the current strategy and the selection of noise, select actions and execute a_t_ = u (s_t_|θ^u^) to obtain the next step status s_t+1_    Store tuple (s_t_,a_t_,r_t_,s_t+1_) to experience replay set D    Calculate reward value r through MFD theory and environmental feedback    Randomly select m samples from replay memory    y_t_=r_t_+ γQ’(s_t+1_, u’(s_t+1_|θ^u’^)|θ^Q’^)  Update current Critical network:$L=\frac{1}{N}\sum_{t}y_{t}{\left(-Q\left(s_{t},a_{t}|\theta^{Q}\right)\right)}^{2}$  Update current Actor network:$\nabla_{\theta^{u}}J\approx \frac{1}{N}\sum_{t}\nabla_{a}{\left.Q\left(s,a|\theta^{Q}\right)\right|}_{s=s_{t},a=u(s_{t})}\nabla_{\theta^{u}}{\left.\left(s|\theta^{u}\right)\right|}_{s_{t}}$  Update target network:θ^Q’^←τθ^Q^+(1-τ)θ^Q’^θ^u’^←τθ^u^+(1-τ)θ^u’^
  **end**
**end**

## 3. Numerical Simulation and Result Analysis

### 3.1. Experimental Setup

**Figure 3:**
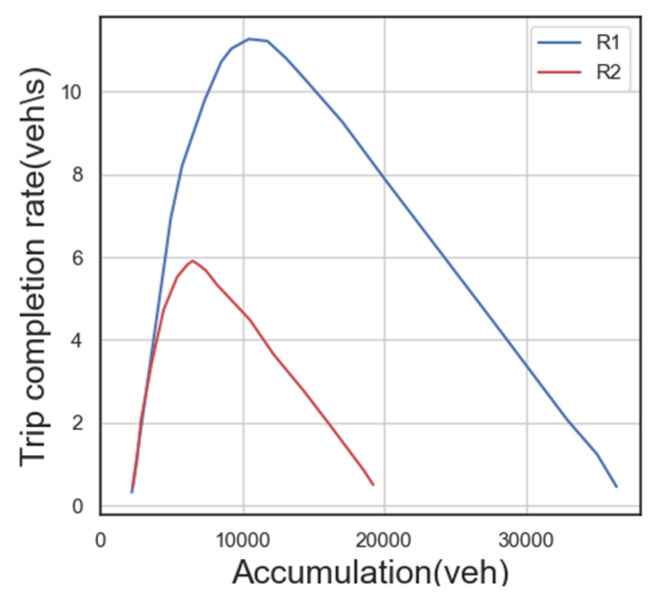
Comparison of MFD models of two traffic districts.

The experimental setup involves two adjacent traffic areas, as depicted in Figure 3. The MFD is used to establish the relationship between traffic demand and the trip completion rate. The chosen MFD diagram corresponds to the one described in previous literature [11]. The basic map represents the MFD of area $R_{1}$, while area $R_{2}$ is scaled down by a certain ratio. The critical traffic volumes for both areas to achieve maximum traffic income are determined as $n_{1,cri}=11,720$ vehicles and $n_{2,cri}=5860$ vehicles.

### 3.2. Parameter Setting

#### 3.2.1. QL Parameter Setting

State Space S: The state space includes the real-time weighted average traffic speed of the traffic area and the remaining duration of the current traffic phase.

Action Space A: The action space of the agent can be defined as Formula (7):

(7) $$\langle a_{(e-w),s\wedge r},a_{(e-w),l},a_{(s-n),s\wedge r},a_{(s-n),l}\rangle$$

In Formula (7), $a_{(e-w),s\wedge }{}_{r}$ represents the phase-switching action of going straight or turning right in the east–west direction, $a_{(e-w),l}$ represents the phase-switching action of turning left in the east–west direction, $a_{(s-n),s\wedge }{}_{r}$ represents the phase-switching action of going straight or turning right in the north–south direction, and $a_{(s-n),}{}_{l}$ represents the phase switching action for turning left in a specific direction.

The reward function is as Formula (8):

(8) $$R_{i}(t)=\left\{\begin{array}{c} \sum_{all-edges}accu\_travel\_time,p_{i}\in p_{i1}(t)\\ \sum_{all-edges}accu\_travel\_time\cdot \eta ,p_{i}\in p_{i2}(t) \end{array}\right.$$

In Equation (9), when the traffic state of the traffic area falls into an oversaturated state, at this point, a proportional coefficient will be used to punish the road network for falling into an oversaturated state when receiving rewards η:

(9) $$\eta =\frac{V_{i}(t)}{V_{cri}},\eta \in (0,1]$$

#### 3.2.2. DDPG Parameter Setting

State space S: For each agent, the state includes four vehicle accumulations: $n_{11}(t)$, $n_{12}(t)$, $n_{21}(t)$, and $n_{22}(t)$, and four traffic demands: $q_{11}(t)$, $q_{12}(t)$, $q_{21}(t)$, and $q_{22}(t)$. These values are normalized and scaled to the interval $\left[0,1\right]$ by taking the maximum value as the reference.

Action space A: For the agent, its action is determined by two values in the selectable range $\left[u_{\min},u_{\max}\right]$ of the perimeter controllers $u_{12}$ and $u_{21}$.

Reward function R: The training objective of the agent is to maximize the cumulative number of vehicle trips completed. The reward is defined as $(M_{11}(t)+M_{22}(t))/B$, where B is a constant, and the rewards are normalized to $\left[0,1\right]$.

### 3.3. Result Analysis

#### 3.3.1. Convergence Analysis

**Figure 4:**
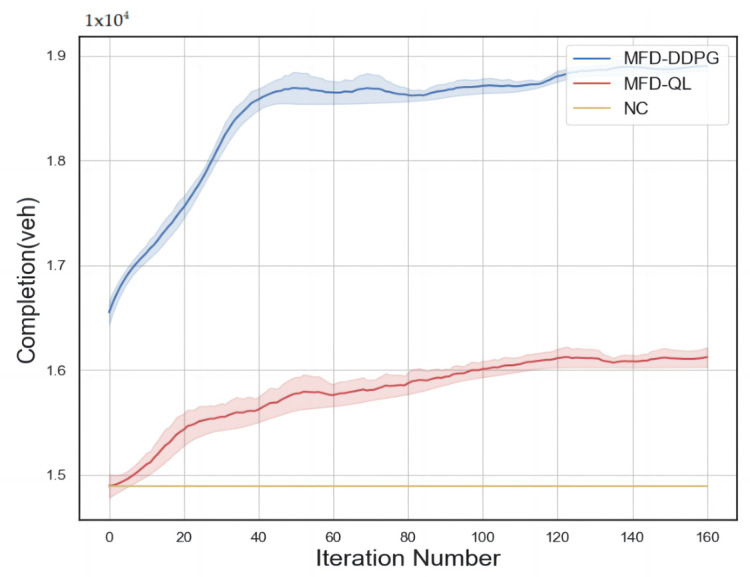
Performance comparison of control strategies.

The performance curves of the No Control strategy, MFD-QL perimeter control strategy, and MFD-DDPG control strategy from the numerical simulation experiment are presented in Figure 4. The horizontal axis represents the number of iterations in the numerical simulation generator of the simulation platform, while the vertical axis represents the cumulative number of completed vehicle trips. The shaded area of the curve indicates the two extreme value intervals in each iteration, representing the inherent randomness of the agent’s learning process.

From Figure 4, it can be observed that both perimeter control models exhibit continuous learning capabilities within the numerical simulation environment and gradually converge over time. They demonstrate good convergence properties. Comparing the two models, the MFD-DDPG perimeter control model proves to be more effective in addressing the perimeter control problem based on the MFD.

#### 3.3.2. Effectiveness Analysis

**Figure 5:**
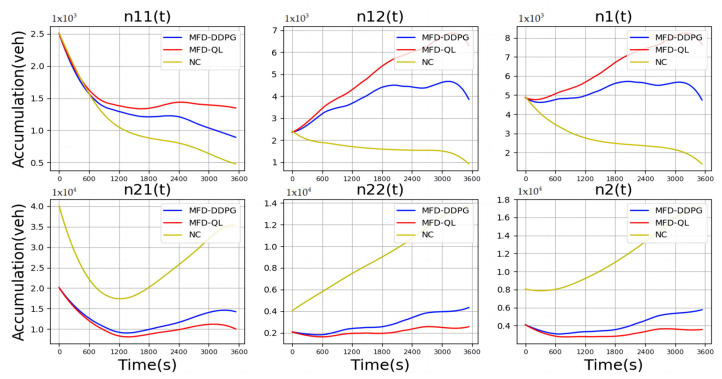
Comparison of cumulative vehicle trends.

Figure 5 presents the evolution trend diagram of vehicle accumulation in the traffic state quantity. It can be observed that both the MFD-QL perimeter control model and the MFD-DDPG perimeter control model effectively prevent the traffic area, which initially starts in an unsaturated state, from falling into an oversaturated state. Additionally, these models improve the traffic income in such areas. Moreover, for the traffic area initially in an oversaturated state, the models successfully alleviate traffic congestion and maintain traffic income in an unsaturated state. These results highlight the effectiveness of the MFD-QL and MFD-DDPG perimeter control models in optimizing traffic control and managing traffic congestion.

**Figure 6:**
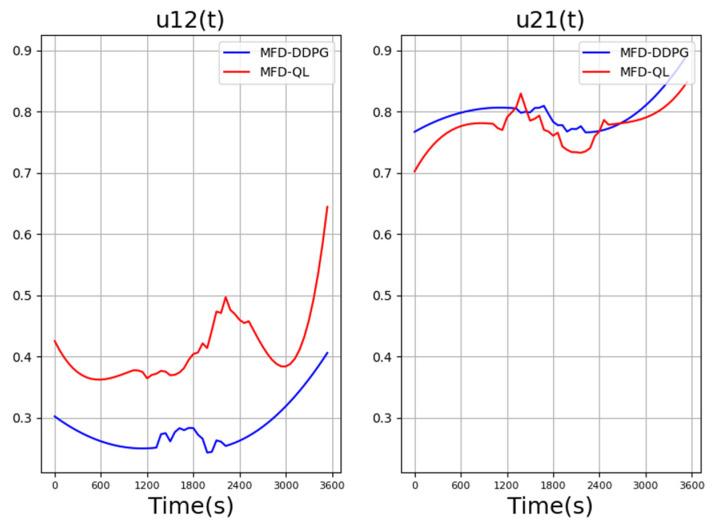
Comparison of perimeter controller values in MFD-QL and MFD-DDPG perimeter control models.

Figure 6 compares the values of the perimeter controllers in the last iteration of the MFD-QL perimeter control model and the MFD-DDPG perimeter control model. It can be observed that the reward values of the perimeter controllers in both models exhibit similar changing trends.

However, when faced with changes in traffic demand, the range of action changes in the MFD-DDPG perimeter control model is smaller compared to the perimeter controller in the MFD-QL perimeter control model.

## 4. Simulation Experiment and Result Analysis

### 4.1. Experimental Setup

**Figure 7:**
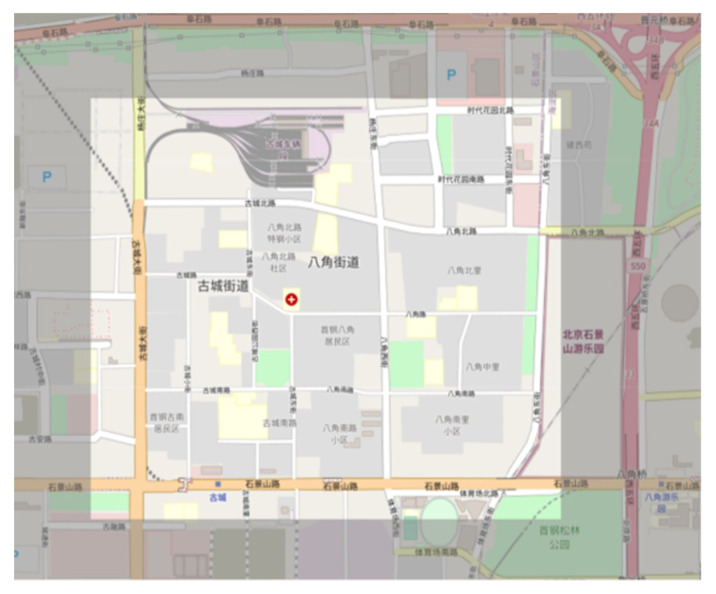
Bajiao street road network map.

**Figure 8:**
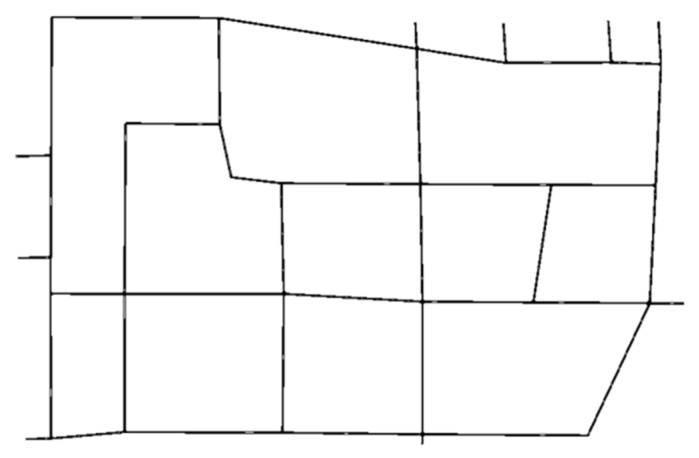
SUMO simulation model of Bajiao street regional road network.

The traffic area intercepted by the traffic simulation tool SUMO is shown in Figure 7, specifically the area enclosed by Gucheng North Road, Bajiao North Road, Bajiao East Street, Shijingshan Road, and Gucheng Street. The base drawing SUMO-GUI in Figure 8 shows the road network.

Table 2 and Table 3 show the traffic information of the controlled intersections.

Table 4 shows the traffic flow of the surveyed upstream sections during peak hours (07:00–09:00, 17:00–19:00) and use this data as input for simulation data. 

### 4.2. Parameter Setting

Both perimeter control models use the above test road network, so the key element settings are the same.

#### 4.2.1. Environmental State Design

Typically, there are two types of state data in the traffic environment: static data and dynamic data. Static data are data that can remain constant for a certain period of time within a signal cycle. Dynamic data are data that change dynamically in real time with the simulation step. By incorporating these two types of data, the control models can effectively capture and respond to the current traffic conditions, enabling informed decision making and the optimization of the signal control strategy. Formula (10) represents the environmental state:

(10) $$s_{t}{}^{j}=\left\{p_{t}{}^{j},v_{t}{}^{1},\cdot \cdot \cdot ,v_{t}{}^{n}\right\}$$

Among them, state $s_{t}{}^{j}$: the traffic state of the intersection corresponding to agent j at time t; phase number $p_{t}{}^{j}$: the phase number of the signal light at the intersection corresponding to agent j at time t; average lane speed $v_{t}{}^{n}$: lane n at time t average speed.

#### 4.2.2. Action Design for the Intelligent Agent

The actions and action sets are defined in Formula (11):

(11) $$A^{j}=\left\{a_{1}{}^{j},a_{2}{}^{j},\cdot \cdot \cdot ,a_{n}{}^{j}\right\}$$

Different action sets are designed for each controlled intersection, as shown in Table 5.

#### 4.2.3. Reward Function Design

The definition of the reward is shown in Formula (12):

(12) $$r_{t}{}^{j}=\sum_{n=1}^{n}\frac{1}{w_{n}}$$

Among them, reward $r_{t}{}^{j}$ refers to the reward value of agent j at time t, and “traffic information $\omega_{n}$” refers to the average waiting time of vehicles in lane n at time t.

### 4.3. Result Analysis

In the simulation experiment, the MFD-QL perimeter control model, MFD-DDPG perimeter control model, and fixed timing control were used to simulate the test road network. Convergence analysis was selected as the criterion for evaluating the learning ability of the two models. By comparing the performance of the model in terms of average travel time, average loss time, and average waiting time, the effectiveness and efficiency of the MFD-QL perimeter control model and the MFD-DDPG perimeter control model can be evaluated and compared with a fixed timing control strategy.

#### 4.3.1. Convergence Analysis

**Figure 9:**
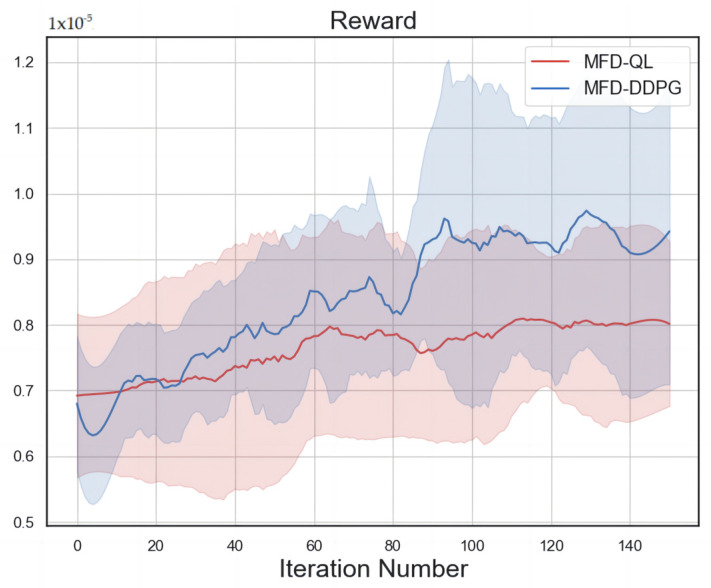
Convergence trend comparison.

The reward value convergence curves of the MFD-QL perimeter control model and the MFD-DDPG perimeter control model are shown in Figure 9, and the shaded part of the curve is formed by the area between the filled mean and variance during each training process. Both the MFD-QL perimeter control model and the MFD-DDPG perimeter control model have continuous learning ability and good convergence ability in the actual road network simulation experiment. The MFD-DDPG perimeter control model exhibits better learning ability and convergence.

A comparison of the convergence curves of the two control models shows that the MFD-DDPG perimeter control model has better learning ability and convergence. In the first 20 trainings, the reward value of the MFD-DDPG perimeter control model is lower than that of the MFD-QL perimeter control model because the MFD-DDPG perimeter control model gives up some data during the training process. However, after 20 training sessions, the reward value of the MFD-DDPG perimeter control model starts to be higher than that of the MFD-QL perimeter control model, and will remain so until the convergence stabilizes. This is because the Q-learning algorithm is not capable of handling high-dimensional data in the face of complex traffic environments, and the DDPG algorithm can better deal with the dimension explosion problem, so MFD-DDPG has a stronger learning efficiency and convergence ability.

#### 4.3.2. Effectiveness Analysis

**Figure 10:**
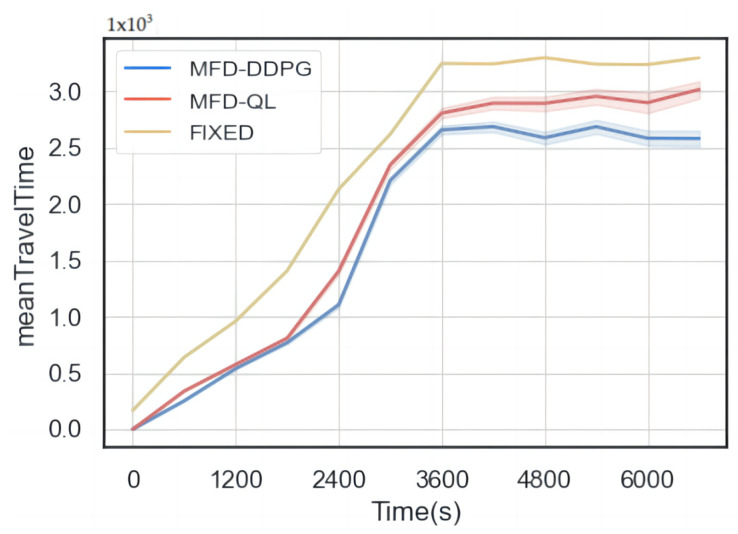
Comparison of average travel time.

**Figure 11:**
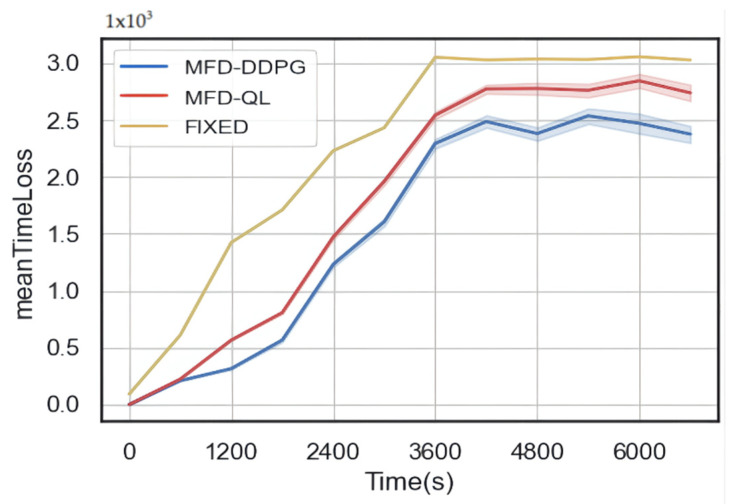
Comparison of average loss time.

**Figure 12:**
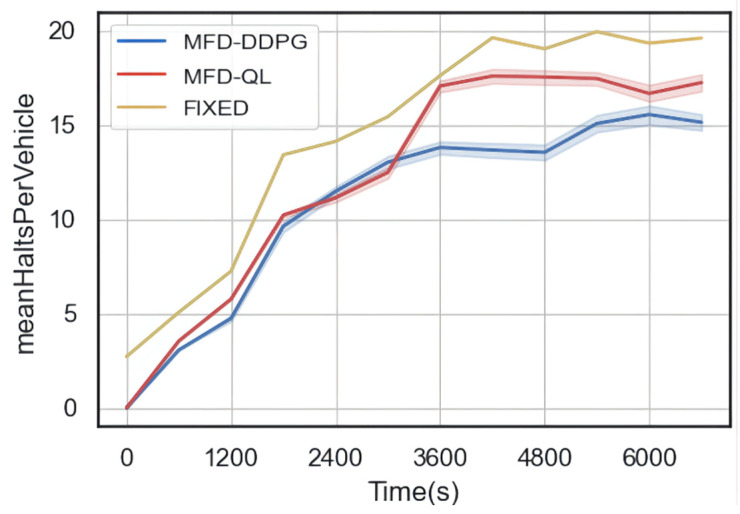
Comparison of average waiting vehicles.

The results of the simulation (Figure 10, Figure 11 and Figure 12) show that both the MFD-QL perimeter control model and the MFD-DDPG perimeter control model outperform the fixed timing control strategy in terms of average travel time, average loss time, and average number of waiting vehicles.

According to Figure 10 and Figure 11, compared to the fixed timing control, both the MFD-QL perimeter control model and the MFD-DDPG perimeter control model demonstrate the ability to reduce the average travel time and average time loss in the test road network. Additionally, according to Figure 12, they can maintain a lower average number of waiting vehicles compared to the fixed timing control strategy. Notably, the MFD-DDPG perimeter control model performs better in these respects.

In summary, both the MFD-QL perimeter control model and the MFD-DDPG perimeter control model contribute to improving the traffic revenue of the test road network. When comparing the two control models, the MFD-DDPG perimeter control model exhibits better control performance and is more effective in handling high-dimensional data.

## 5. Discussion and Conclusions

This article presents a study on deep reinforcement learning in urban traffic area control. Based on the MFD attributes that can characterize the traffic area, the perimeter control problem of the traffic area is proposed, and the control objectives and constraints are clearly defined.

By utilizing the good adaptability of reinforcement learning and deep reinforcement learning in dealing with traffic environments, two different perimeter control models based on deep reinforcement learning were designed according to the perimeter control objective problem, and specific reinforcement learning elements and algorithm processes were designed. Finally, an experimental platform was established to verify the rationality and effectiveness of the proposed perimeter control model.

Through numerical simulation experiments, it was verified that the MFD-QL perimeter control model and MFD-DDPG perimeter control model have good convergence and control effects in numerical simulation experiments, and the two perimeter control models under numerical simulation can also achieve the function of alleviating traffic congestion. Finally, through traffic simulation experiments on actual road networks, it was verified that the MFD-QL perimeter control model and the MFD-DDPG perimeter model can achieve better traffic returns compared to fixed timing control, and also verified that the MFD-DDPG perimeter control has the best control effect.

However, for large and complex urban transportation networks, the model mentioned in the article does not yet achieve good results in terms of applicability; for situations where the transportation network is unstable, other methods need to be found to resolve the problems. In future work, we will analyze recurrent, non-recurrent, and emergency-triggered traffic congestion types, and separately model them to validate the effectiveness of the method proposed in the article.

Ultimately, research opportunities are multifaceted, and we believe that addressing these issues is crucial in order to better address traffic congestion and ensure the greatest traffic benefits.

## Figures and Tables

**Table 1:** Traffic characteristics of traffic district.

Traffic Parameters	Illustrate
i	Traffic area
$S_{i}$	Link collection in the traffic area i
k	A link in the traffic area i
$n_{i}(t)$	The number of vehicles in the traffic area i at a time t
$n_{k}(t),k\in U_{i}$	The traffic volume of the link j at time t
$l_{k}$	The length of a link j
$L_{i}$	The sum of the lengths of all links in the traffic area i
$D_{i}(t)$	The average traffic density of the traffic area i at the moment t
$Q_{i}(t)$	The weighted traffic flow by Formula (2) of traffic area *i* at time t
$H_{i}$	Traffic benefits of traffic area i

**Table 2:** Basic traffic information of controlled intersections.

Control Intersection Location	Intersection ID	Traffic Phase	Signal Period (s)
Gucheng North Road—Gucheng Street	J0	Four-phase fixed timing	116
Gucheng North Road—Bajiao West Street	J1	Four-phase fixed timing	102
Bajiao North Road—Bajiao East Street	J2	Four-phase fixed timing	105
Bajiao South Road—Bajiao East Street	J3	Four-phase fixed timing	115
Shijingshan Road—Bajiao East Street	J4	Four-phase fixed timing	112
Shijingshan Road—Bajiao West Street	J5	Four-phase fixed timing	111
Shijingshan Road—Gucheng Street	J6	Four-phase fixed timing	117
Gucheng Street—Gucheng West Road	J7	Four-phase fixed timing	110

**Table 3:** Fixed timing parameters for controlled intersections.

Control Intersection Location	Phase 1 (s)	Phase 2 (s)	Phase 3 (s)	Phase 4 (s)	Yellow Light (s)
Gucheng North Road—Gucheng Street	36	15	38	15	3
Gucheng North Road—Bajiao West Street	31	16	29	14	3
Bajiao North Road—Bajiao East Street	33	15	32	17	2
Bajiao South Road—Bajiao East Street	36	14	38	15	3
Shijingshan Road—Bajiao East Street	34	14	36	15	3
Shijingshan Road—Bajiao West Street	33	14	36	16	3
Shijingshan Road—Gucheng Street	35	17	38	15	3
Gucheng Street—Gucheng West Road	32	15	35	16/	3

**Table 4:** Traffic flow during peak hours.

Control Intersection Location	Number of Cars (Morning Peak)	Number of Vehicles Arriving (Afternoon Peak)	Total Vehicles
Gucheng North Road—Gucheng Street	1147	1241	2388
Gucheng North Street—Bajiao West Street	1283	1078	2370
Bajiao North Road—Bajiao East Street	928	816	1744
Bajiao South Road—Bajiao East Street	1306	1233	2539
Shijingshan Road—Bajiao East Street	1028	986	2014
Shijingshan Road—Bajiao West Street	1467	1298	2765
Shijingshan Road—Gucheng Street	1239	1083	2322
Gucheng Street—Gucheng West Road	825	921	1746

**Table 5:** Action definition.

Intersection Serial Number	$a_{1}^{j}$	$a_{2}^{j}$	$a_{3}^{j}$	$a_{4}^{j}$
J0	switch to north–south phase, phase duration 30 s	switch to north–south phase, phase duration 15 s	switch to east–west phase, phase duration 20 s	switch to east–west phase, phase duration 10 s
J1	switch to north–south phase, phase duration 30 s	switch to north–south phase, phase duration 15 s	switch to east–west phase, phase duration 20 s	switch to east–west phase, phase duration 10 s
J2	switch to north–south phase, phase duration 25 s	switch to north–south phase, phase duration 15 s	switch to east–west phase, phase duration 20 s	switch to east–west phase, phase duration 15 s
J3	switch to north–south phase, phase duration 30 s	switch to north–south phase, phase duration 20 s	switch to east–west phase, phase duration 20 s	switch to east–west phase, phase duration 15 s
J4	switch to north–south phase, phase duration 25 s	switch to north–south phase, phase duration 15 s	switch to east–west phase, phase duration 20 s	switch to east–west phase, phase duration 15 s
J5	switch to north–south phase, phase duration 30 s	switch to north–south phase, phase duration 25 s	switch to east–west phase, phase duration 25 s	switch to east–west phase, phase duration 20 s
J6	switch to north–south phase, phase duration 20 s	switch to north–south phase, phase duration 15 s	switch to east–west phase, phase duration 20 s	switch to east–west phase, phase duration 15 s
J7	switch to north–south phase, phase duration 35 s	switch to north–south phase, phase duration 15 s	switch to east–west phase, phase duration 20 s	switch to east–west phase, phase duration 25 s

## Data Availability

Not applicable.
